# Machine learning based gray-level co-occurrence matrix early warning system enables accurate detection of colorectal cancer pelvic bone metastases on MRI

**DOI:** 10.3389/fonc.2023.1121594

**Published:** 2023-03-22

**Authors:** Jinlian Jin, Haiyan Zhou, Shulin Sun, Zhe Tian, Haibing Ren, Jinwu Feng, Xinping Jiang

**Affiliations:** Gezhouba Central Hospital of Sinopharm, The Third Clinical Medical College of China Three Gorges University, Yichang, Hubei, China

**Keywords:** colorectal cancer, bone metastasis, gray-level co-occurrence matrix, machine learning, prediction

## Abstract

**Objective:**

The mortality of colorectal cancer patients with pelvic bone metastasis is imminent, and timely diagnosis and intervention to improve the prognosis is particularly important. Therefore, this study aimed to build a bone metastasis prediction model based on Gray level Co-occurrence Matrix (GLCM) - based Score to guide clinical diagnosis and treatment.

**Methods:**

We retrospectively included 614 patients with colorectal cancer who underwent pelvic multiparameter magnetic resonance image(MRI) from January 2015 to January 2022 in the gastrointestinal surgery department of Gezhouba Central Hospital of Sinopharm. GLCM-based Score and Machine learning algorithm, that is,artificial neural net7work model(ANNM), random forest model(RFM), decision tree model(DTM) and support vector machine model(SVMM) were used to build prediction model of bone metastasis in colorectal cancer patients. The effectiveness evaluation of each model mainly included decision curve analysis(DCA), area under the receiver operating characteristic (AUROC) curve and clinical influence curve(CIC).

**Results:**

We captured fourteen categories of radiomics data based on GLCM for variable screening of bone metastasis prediction models. Among them, Haralick_90, IV_0, IG_90, Haralick_30, CSV, Entropy and Haralick_45 were significantly related to the risk of bone metastasis, and were listed as candidate variables of machine learning prediction models. Among them, the prediction efficiency of RFM in combination with Haralick_90, Haralick_all, IV_0, IG_90, IG_0, Haralick_30, CSV, Entropy and Haralick_45 in training set and internal verification set was [AUC: 0.926,95% CI: 0.873-0.979] and [AUC: 0.919,95% CI: 0.868-0.970] respectively. The prediction efficiency of the other four types of prediction models was between [AUC: 0.716,95% CI: 0.663-0.769] and [AUC: 0.912,95% CI: 0.859-0.965].

**Conclusion:**

The automatic segmentation model based on diffusion-weighted imaging(DWI) using depth learning method can accurately segment the pelvic bone structure, and the subsequently established radiomics model can effectively detect bone metastases within the pelvic scope, especially the RFM algorithm, which can provide a new method for automatically evaluating the pelvic bone turnover of colorectal cancer patients.

## Introduction

Worldwide, colorectal cancer is still a malignant tumor of the digestive system with a high incidence rate and mortality (1). In recent years, it is encouraging that advanced diagnostic technologies, such as computed tomography (CT) colon imaging, magnetic resonance imaging (MRI) and positron emission tomography (PET)/CT colon imaging, are beneficial to enable some early cancer patients to receive timely treatment and effectively reduce the recurrence and metastasis rate (2, 3). Nevertheless, the poor prognosis caused by colorectal cancer metastasis is still one of the important factors that can not be ignored and avoided.

Previous studies have focused on the common metastatic sites of colorectal cancer, including lymph node metastasis, liver metastasis, and so on (4–6). Vigilantly, bone metastasis is also a poor prognostic factor for colorectal cancer, with incidence rate ranging from 2% to 11% (7, 8). As one of the advanced diseases of colorectal cancer, due to the heterogeneity and complexity of bone metastasis, there are great differences in the survival and recurrence of patients (9–11). Previous studies have shown that the most common sites of bone metastasis in colorectal cancer patients are the pelvis, thoracic vertebrae and lumbar vertebrae, while there may be metastatic or implanted small lesions in the pelvis and the pelvic cavity near the sacrum. Cancer emboli can be directly transferred to the pelvis, or transferred to the sacrum through the capillaries of the sacrum, leading to vertebral bone metastasis or other sites (9–11). Therefore, it is urgently needed that the available prediction model can divide patients into different categories according to the risk score of pelvic bone metastasis, so as to select appropriate treatment methods, and can also more accurately evaluate the effectiveness of treatment measures.

Nowadays, radiomics and advanced algorithms have been gradually applied to the medical field, of which the most widely used is clinical prediction model (12, 13). Gray level co-occurrence matrix(GLCM) has been widely used in disease diagnosis, clinical staging, treatment evaluation and prognosis evaluation (14, 15). With the help of the spatial correlation characteristics of gray level, the modified technology can describe the image texture, which can efficiently extract and model the features of a variety of medical images (16, 17). Additionally, the higher-order algorithm of machine learning, with its iterative weight distribution, can make better use of predictors to improve the diagnostic efficiency of the model.

Inspired by this, this study based on diffusion weighted imaging, on the basis of applying machine learning algorithm to automatically segment the pelvic bone structure, established radiomics model to judge whether there is bone metastasis in the pelvic bone structure of patients with colorectal cancer, in order to better serve clinical decision-making.

## Materials and methods

### Study population

**Figure 1:**
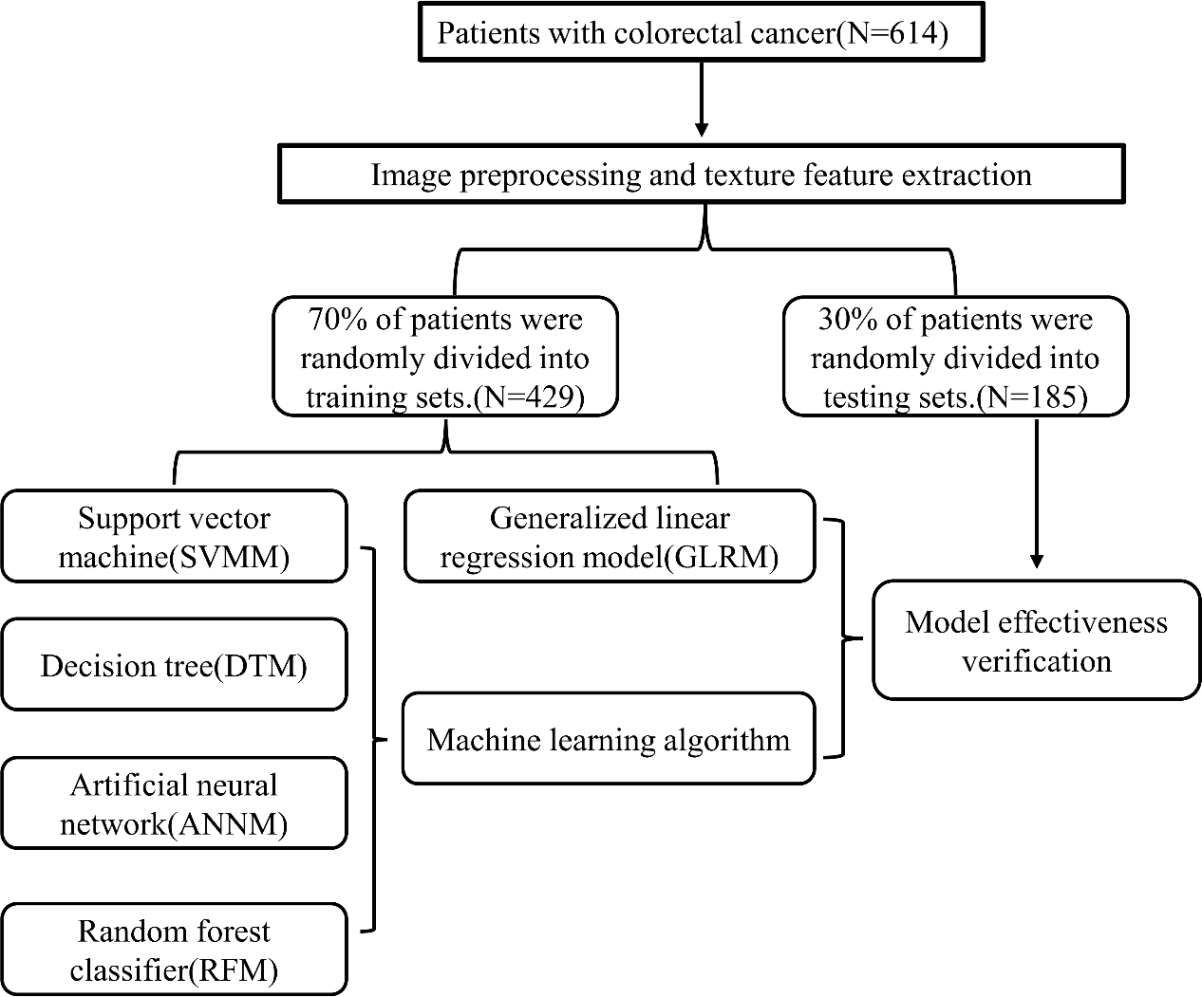
The flow chart of patient selection and data process.

We retrospectively included 614 patients with colorectal cancer who underwent pelvic multiparameter MRI from January 2015 to January 2022 in the gastrointestinal surgery department of Gezhouba Central Hospital of Sinopharm. Comparison against abdominopelvic CT, SPECT/CT, or histopathologic tissue sampling was used to establish the baseline ground truth for presence or absence of bone metastases at the time of enrollment. The inclusion criteria of patients are as follows: (i)Patients suspected of colorectal cancer or undergoing pelvic multi-parametric diffusion weighted imaging(mp-DWI) scan due to reexamination after colorectal cancer treatment; (ii)Patients with complete pelvic DWI images; (iii)Patients without primary pelvic bone disease (primary osteosarcoma, bone cyst, blood system disease, fracture, etc.). Exclusion criteria: (i)Patients with a history of pelvic bone structure surgery; (ii)Patients with a history of other malignancies; (iii)Patients with unsatisfied image quality, such as motion artifacts and chemical shift artifacts; (iv)Patients with incomplete scanning scope and not including most pelvic bone structures. This retrospective study was approved by the Ethics Committee of Gezhouba Central Hospital of Sinopharm, and the research scheme was implemented according to the artificial intelligence(AI) model training specifications of the unit. All patients’ personal information is encrypted to prevent leakage, and complies with the Declaration of Helsinki. All patients in this study were informed of the study protocol and approved the study by written consent. The process of incorporating patients and building prediction models was shown in **Figure 1**.

### Acquisition of diffusion weighted imaging parameters

We used GE Discovery MR750 W3.0 T machine to perform pelvic MRI plain scan and enhanced scan. The patient was instructed to lie on his back with his head advanced, and the scanning range was from the umbilical foramen level to the pubic symphysis. The sequences included conventional transverse T1WI, (with or without) conventional transverse T2WI, (transverse, sagittal, coronal) fat compression T2WI, (with or without sagittal) transverse DWI (b value=1000s/mm, b value=2000 s/mm), transverse and sagittal fat compression LAVA enhanced sequences. Sagittal fat compression T2WI scanning parameters: TR 4 800.0 ms, TE 110.5 ms, matrix 320 × 320, layer thickness and layer spacing are 5 mm and 1 mm respectively, FOV range: 28 cm × 28 cm.

Next, we standardized the format of DWI, that is, converted the high b value DWI image in DICOM format to Nifty format, and then the radiology resident (with film reading experience of 3 years or more) used ITK-SNAP3.6.0 software(http://www.itksnap.org/pmwiki/pmwiki.php?n=Main.Publications ) to manually delineate and label the DWI image along the edges of various pelvic bone structures. In addition, a radiologist (with film reading experience ≥ 15 years) modified and confirmed the label, and the confirmed image label was used as the gold standard of pelvic bone structure segmentation model.

### Training and verification of segmentation model

We preprocessed the image and extract the texture features, and used the software GE (Shanghai) AK (Artifical Intelligence Kit, V3.2.0R version) application platform to preprocess the image: linear method is used for resampling, with X, Y, Z spacing of 1.000; Gaussian 0.50 is used for noise removal; MR bias field correction is adopted to eliminate the stray intensity change caused by the non-uniformity of magnetic field and coil; Intensity standardization adopts gray discretization, and the expected minimum value and maximum value are 0.015 and 0.255,respectively. After image preprocessing (resampling, offset field correction, intensity standardization), a total of 48 GLCM texture features are finally collected by AK software, including 8 types of parameters, namely: cluster facilitation, cluster shade, correlation, GLCM energy, GLCM entropy, Haralick correlation, inertia, inverse difference motion; 6 angles are calculated, including full angle, full angle SD, 0°, 45°, 90° and 135°.

### Analysis and evaluation of pelvic bone metastasis prediction model

We preprocessed the DWI images of 614 patients, namely: size=64 × 224 × 224 (z, y, x), automatic window width and level. Patients were randomly divided into training set (70%) and verification set (30%) according to 7:3. In order to eliminate the imbalance of the classified training set data, we balance the positive/negative samples by reducing the sampling, and use Min Max to normalize the feature matrix. At the same time, we use Pierce correlation coefficient to reduce the dimension of the data, and the eigenvectors of the transformed eigenmatrix have independent features.

There are four machine learning model building methods used in this study, including artificial neural network model (ANNM), random forest model(RFM), decision tree model(DTM) and support vector machine model (SVMM) (18–21). As linear regression models are often used to build clinical prediction models, this study builds generalized linear regression model (GLRM) based on Softmax regression (22, 23), namely: $\phi k=1-\sum_{i-1}^{k-1}\emptyset i$ RFM, DTM, ANNM and SVM are the most commonly used algorithms in machine learning (18). In this study, a pelvic bone metastasis prediction model was built based on four supervised learning algorithms.

Before building the prediction model, we use recursive feature elimination algorithm to select features and sort them, and select the first 8 features as the best feature subset; At the same time, for the GLRM, the minimum absolute shrinkage and selection operator classifier are selected to establish a classification model for predicting pelvic bone metastasis based on DWI images (24). The effectiveness evaluation of each model mainly includes decision curve analysis(DCA) (25), area under the receiver operating characteristic (AUROC) curve and clinical influence curve(CIC) (26).

### Statistical methods

The distribution of “measurement” and “counting” data in accordance with normal distribution in this study is expressed by means (interquartile interval) and percentage (%). For the independent two sample nonparametric test, the Mann Whitney rank sum test is used for the inter-group comparison that does not meet the normal distribution (27); The t-test or chi square test is used for the inter group comparison of samples from normal or nearly normal populations. In addition, the visual analysis of all charts in this study was completed with R studio software (download website: https://www.r-project.org/); Two tailed P values less than 0.05 were considered statistically significant.

## Results

### Patient baseline data and image segmentation characteristics

According to *Caret* software package algorithm, 614 patients included in this study were randomly divided into training set (N=429,70%) and internal verification set (N=185,30%) according to 7:3. The clinical characteristics and image data sources of patients in the data set were summarized in **Table 1** and **Supplementary Table 1**. The average age of patients used for pelvic bone structure segmentation model training was 58 (50, 68) years. Among all the patients used to establish the pelvic bone metastasis classification histological model, 53 patients had bone metastasis [average age 55 (47, 65) years], and 561 patients had no bone metastasis [average age 58 (50, 68) years]. In addition, in the split model sample group, there was no statistically significant difference in clinical characteristics (age, pathology, osseous alteration, CEA and tumor location) between the training set and the internal validation set (P>0.05); The three types of GLCM parameters, namely correlation (full angle, 0°, 45°, 90°), inertia (full angle, full angle SD, 0°, 45°, 90°), inverse difference (full angle, 0°, 45°, 90°), cluster prominence and cluster shadow, had statistical differences (P<0.05), while energy, entropy and Haralick correlation had no statistical differences (P>0.05).

**Table 1:** Baseline data of patients with colorectal cancer.

Variables	Overall (N=614)
Age (median [IQR]),year	58.00 [49.25, 68.00]
sex (%)	
Male	381 (62.1)
Female	233 (37.9)
Pathology (%)	
Adenocarcinoma	293 (47.7)
Squamous cell carcinoma	231 (37.6)
Adenosquamous carcinoma	61 (9.9)
Small cell carcinoma	29 (4.7)
Tumor stage (%)	
I-II	533 (86.8)
III	39 (6.4)
IV	42 (6.8)
Differentiation (%)	
High	326 (53.1)
Moderate	189 (30.8)
Low	99 (16.1)
OA (%)	
Osteolytic	393 (64.0)
Osteogenic	188 (30.6)
Miscibility	33 (5.4)
CEA (%),ng/mL	
<100	156 (25.4)
≥100	458 (74.6)
Tumor location (%)	
Colonic segment	197 (32.1)
Rectal segment	417 (67.9)
ECOG (%)	
0-2	278 (45.3)
>2	336 (54.7)
EV (median [IQR])	0.96 [0.70, 1.22]
Entropy (median [IQR])	8.63 [8.37, 8.87]
IG_all (median [IQR])	3.06 [2.62, 3.58]
IG_0 (median [IQR])	2.20 [1.84, 2.58]
IG_45 (median [IQR])	2.96 [2.55, 3.39]
IG_90 (median [IQR])	2.30 [1.81, 2.77]
IV_all (median [IQR])	186.50 [159.00, 216.75]
IV_all_SD (median [IQR])	5229.00 [3691.25, 6978.50]
IV_0 (median [IQR])	159.05 [127.47, 193.60]
IV_45 (median [IQR])	159.85 [124.62, 192.50]
IV_90 (median [IQR])	131.00 [108.25, 155.00]
Haralick_all (median [IQR])	0.10 [0.09, 0.10]
Haralick_30 (median [IQR])	0.10 [0.09, 0.11]
Haralick_45 (median [IQR])	0.07 [0.07, 0.08]
Haralick_90 (median [IQR])	0.11 [0.10, 0.13]
CSV (median [IQR])	90.00 [84.00, 96.00]
CP (median [IQR])	79.00 [72.00, 85.00]

### Feature variable screening based on GLCM prediction model

**Figure 2:**
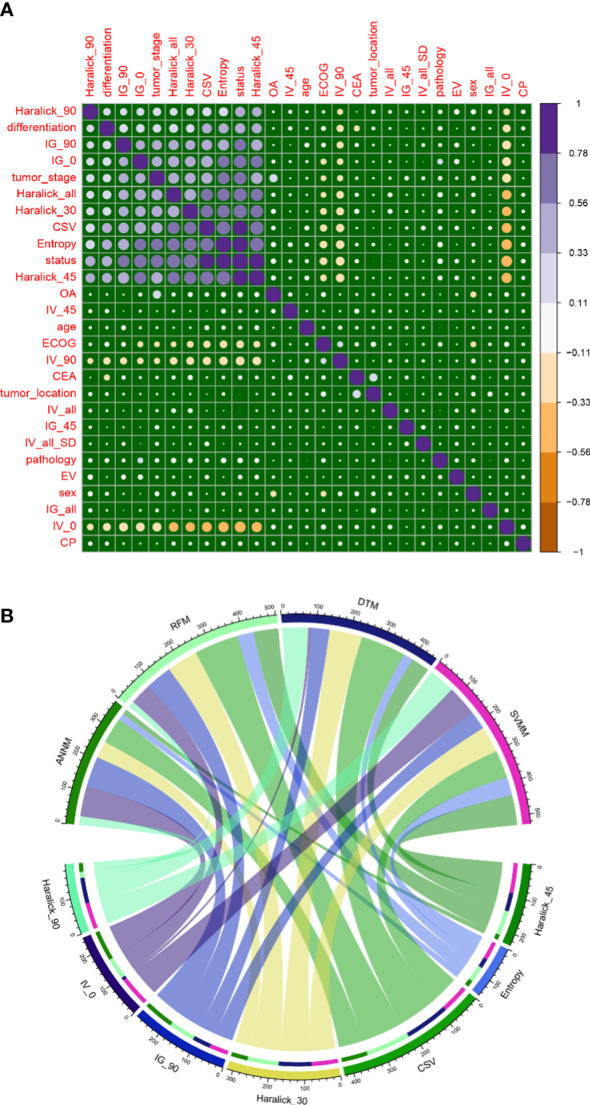
Candidate variables related to pelvic bone metastasis in colorectal cancer. **(A)** Correlation between outcome variables of bone metastasis and candidate variables of GLCM; **(B)** Intersection candidate variables of four prediction models based on machine learning algorithm.

Then, we compared the classification features of bone metastasis lesions based on GLCM between groups, and analyzed the correlation between patients’ baseline data and candidate variables of bone metastasis. The results showed that Haralick_90, Haralick_all, IV_0, IG_90, IG_0, Haralick_30, CSV, Entropy and Haralick_45 were highly positively correlated with pelvic bone metastasis in colorectal cancer patients (**Figure 2A**). In addition, in order to build radiomics model for colorectal cancer patients to conduct classification and evaluation with and without pelvic bone metastasis, we conducted feature extraction from the labeled and segmented images and labels based on the manually labeled and automatically segmented pelvic bone structures, respectively. The extracted features were used to establish the radiomics model, and the processing steps included data equalization, data normalization, feature dimension reduction, and feature selection. As shown in **Figure 2B**, Haralick_90, IV_0, IG_90, Haralick_30, CSV, Entropy and Haralick_45 were the intersection candidate predictors of RFM, SVM, ANNM and DTM.

### Construction of bone metastasis model based on generalized linear algorithm

**Figure 3:**
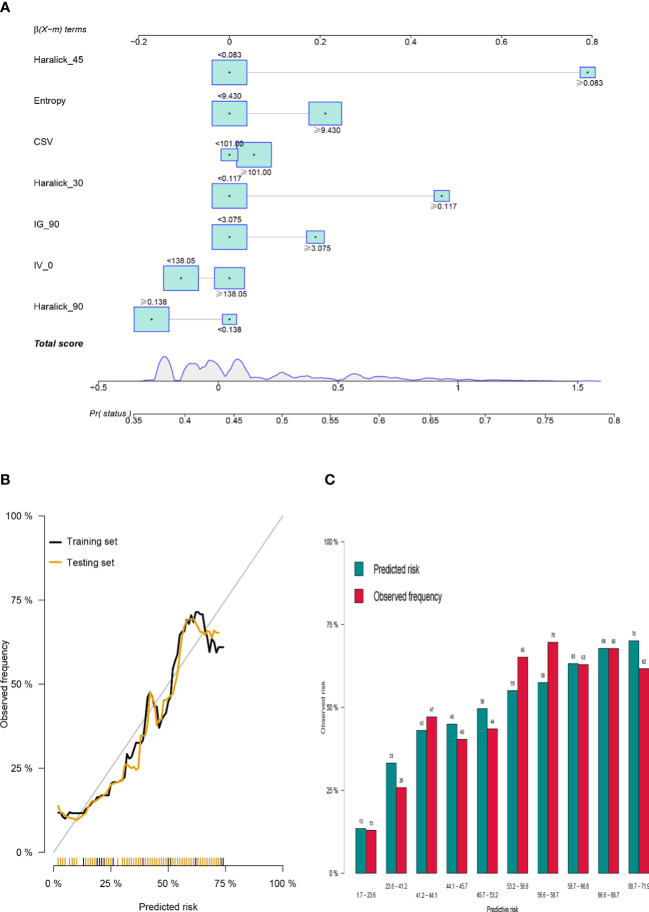
Construction of GLRM to predict bone metastasis in colorectal cancer patients. **(A)** Prediction score of bone metastasis based on Nomogram visualization; **(B)** Robustness evaluation of GLRM in training set and internal test set; **(C)** Multi-sample model prediction verification based on resampling.

According to the results of multiple logistic regression analysis, a nomograph (**Figure 3A**) was developed. The visual quantitative mapping tool of the prediction model was based on the scaling of each regression coefficient to 0 to 100 points in the multiple logistic regression. β The influence of the variable with the highest coefficient (absolute value) is assigned 100 points. Add the scores of all independent variables to get a total, and then convert it into the probability of predicting pelvic bone metastasis. Generally, the C index and AUC value exceeding 0.6 implied a reasonable estimate. This study showed that the C index of GLRM was 0.72, and resampling also showed that the model had an ideal robustness (**Figures 3B, C**).

### Construction of bone metastasis model based on machine learning algorithm

**Figure 4:**
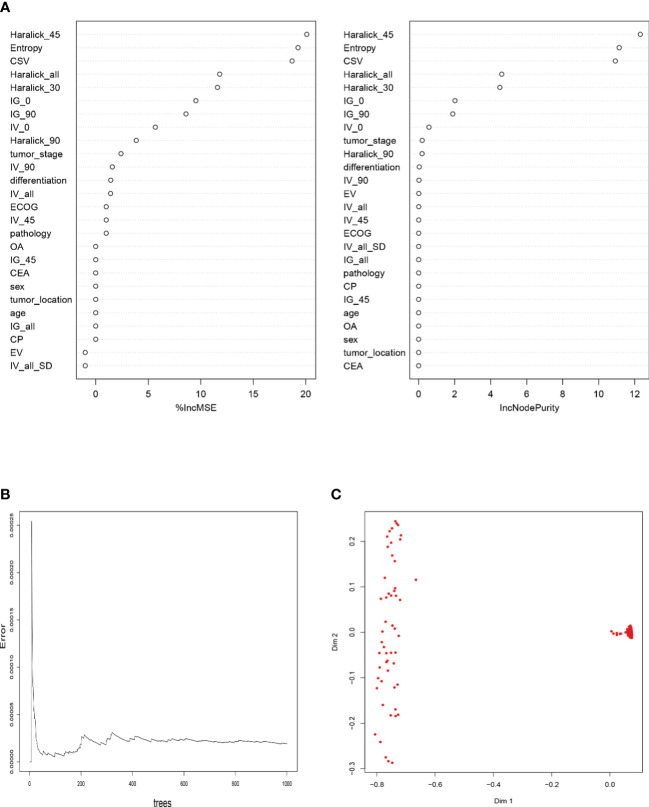
Construction of bone metastasis prediction model of colorectal cancer based on RFM. **(A)** Sorting of RFM prediction variables based on “Pruning” algorithm; **(B)** Screening optimal subset based on ten fold fold cross validation; **(C)** Recognition visualization of RFM in differentiating patients with or without pelvic bone metastasis.

As shown in **Figure 4** and **Supplementary Table 2**, the RFM based on the “bagging” algorithm sorted the GLCM parameters, where Haralick_90, Haralick_all, IV_0, IG_90, IG_0, Haralick_30, CSV, Entropy and Haralick_45 were suitable for further model building of the RFM algorithm; The prediction efficiency of the model showed that the model still had a robust and efficient prediction efficiency (AUC: 0.926,95% CI: 0.873-0.979), even though it passed the ten fold cross validation. Consistent with the parameter variables of the RFM model, DTM (**Supplementary Figure 1**) adopted CSV, Entropy and Haralick_30 as the decision factors in the “branches” of the model, and its prediction efficiency in the training set was worse than that of RFM (AUC: 0.889,95% CI: 0.836-0.942); However, ANNM included eight parameters, namely, Entropy, IG_90, IV_0, IV_45, IV_90, Haralick_al, Haralick_30, Haralick_45, Haralick_90 and CSV. The prediction efficiency obtained was AUC: 0.912, 95% CI: 0.859-0.965, which was worse than RFM, but better than DTM, SVM and GLRM.

### Efficacy evaluation of five bone metastasis prediction models

**Figure 5:**
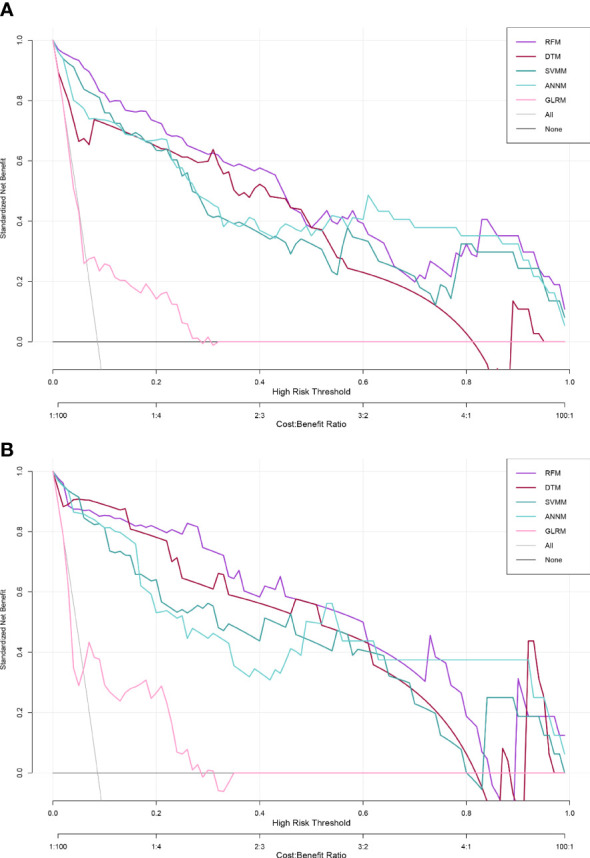
Effectiveness evaluation of five predictive models for pelvic bone metastasis detection based on DCA. **(A)** Training set; **(B)** Internal validation set.

DCA is a relatively new model evaluation method compared with ROC curve (28). In this study, we adopted two model effectiveness evaluation methods. We verified the linear regression model GLRM and four machine learning models (RFM, ANNM, SVM and DTM) with an internal test set. From the perspective of prediction accuracy, we can see that RFM has the largest “net benefit” in DCA(threshold probability=0.81), followed by ANNM, DTM and SVM (**Figure 5** and **Supplementary Table 3**). GLRM was the least predictive machine learning model, with threshold probability=0.54.

At the same time, the ROC curve showed that the diagnostic efficacy of bone metastasis of RFM in training set and verification set was [AUC: 0.926,95% CI: 0.873-0.979] and [AUC: 0.919,95% CI: 0.868-0.970] respectively, while the diagnostic efficacy of bone metastasis of ANNM in training set and verification set was [AUC: 0.912,95% CI: 0.859-0.965] and [AUC: 0.894,95% CI: 0.843-0.945] respectively, which was slightly lower than that of RFM; As shown in **Table 2** and **Supplementary Figure 2**, in general, the prediction efficiency of the prediction model for pelvic metastasis of colorectal cancer constructed by machine learning algorithm was better than that of the traditional generalized linear model. The results were consistent both in the training set and internal test set.

**Table 2:** Comparison of predictive efficacy of five types of pelvic bone metastasis prediction models.

Model	Training set			Internal validation set		
	AUC Mean	AUC 95%CI	Variables^&^	AUC Mean	AUC 95%CI	Variables^&^
RFM	0.926	0.873-0.979	7	0.919	0.868-0.970	7
SVMM	0.862	0.809-0.915	11	0.841	0.790-0.892	11
DTM	0.889	0.836-0.942	5	0.839	0.788-0.890	5
ANNM	0.912	0.859-0.965	10	0.894	0.843-0.945	10
GLRM	0.716	0.663-0.769	7	0.722	0.671-0.773	7

^&^Variables included in the model.

### Prediction effectiveness evaluation of optimal prediction model

Based on the evaluation of five prediction models for pelvic metastasis of colorectal cancer, we found that RFM was the best in terms of prediction efficiency. In order to further evaluate the differentiation efficiency of RFM, we used CIC to evaluate the “classification accuracy” of RFM in training set and internal verification set. As shown in **Supplementary Figure 3**, the blue curve (number high risk with outcome) indicated the number of true positives under each threshold probability, and the red curve (numberhigh risk) indicated the number of people classified as positive (high risk) by the prediction model under each threshold probability. It was credible that RFM can accurately distinguish patients with bone metastasis from those without bone metastasis, whether in training set or internal verification set, which further confirms that RFM can not only increase the interpretability of bone metastasis risk grading model for colorectal cancer patients, but also improve the grading accuracy. Therefore, RFM was suitable for stratified diagnosis and treatment.

## Discussion

Invasion of colorectal cancer has always been a difficult problem in the treatment process. Because cancer cells can metastasize remotely through lymphatic vessels, blood vessels and nerves, especially vascular invasion, that is, cancer cells can metastasize remotely earlier through the portal vein and inferior vena cava (29–31). In the early stage of treatment, pay close attention to the degree of colorectal cancer invasion, and carefully check the metastatic lymph nodes (4, 32). For those with high degree of invasion or lymph node metastasis, prepare radiotherapy and chemotherapy plans in advance. Routine treatment after radical surgery can have a very important guiding value to improve the prognosis of patients. As far as we know, this is the first attempt to integrate machine learning algorithm and imaging information to build a prediction model for colorectal cancer bone metastasis. Compared with previous studies, this study extracts a series of information that cannot be directly observed by the naked eye through quantitative and high-throughput analysis and processing of medical images, which can better reveal the relationship between tumor biological characteristics and images, and can be used to establish descriptive and predictive models to help doctors make clinical decisions.

At present, studies have found that preoperative T staging, lymphatic metastasis and Duckes staging of colorectal cancer are independent factors that affect the prognosis of colorectal cancer (33–35). In addition, anesthesia, perioperative blood transfusion and treatment are also relevant factors. However, most of the above factors are based on liver metastasis and lung metastasis, but there is little analysis on the influencing factors of bone metastasis of colorectal cancer (5, 36). There is also a lack of reliable potential indicators that can predict bone metastasis of colorectal cancer. In view of this, this study strives to explore the main influencing factors of bone metastasis after radical resection of colorectal cancer, explore its potential bone metastasis prediction indicators, and build a bone metastasis prediction model based on advanced algorithms. As far as we know, this is the first prediction model for pelvic bone metastasis of colorectal cancer based on radiomics and machine learning. With the help of this model, we hope to better guide clinical diagnosis and treatment.

The bone metastasis of colorectal cancer is mainly osteogenic lesions, with multiple and jumping distribution, and osteogenic changes and osteolytic changes exist at the same time (37). Fortunately, mpMRI has a high sensitivity and specificity in the diagnosis of colorectal cancer bone metastasis (38). When both systemic bone phenomena and CT cannot determine the existence of bone metastasis, mpMRI is usually feasible. Generally speaking, mpMRI includes conventional sequences (TIW1 and T2W1) and functional sequences (DWI, DCE-MRI and MRS) (38, 39). Among them, DWI is more sensitive to monitoring bone metastasis of colorectal cancer than conventional sequences. DWI is an assessment of microscopic movement of water molecules in the body, and can provide quantitative (such as ADC value) and qualitative (such as signal strength) information for disease diagnosis and treatment.

Radiomics is a new image post-processing technology emerging in recent years. Through quantitative and high-throughput analysis and processing of medical images, it extracts a series of information that cannot be directly observed by the naked eye, reveals the relationship between tumor biological characteristics and images, and is used to establish descriptive and predictive models to help doctors make diagnosis (40). In this study, on the basis of segmentation of pelvic bone structure, we established radiomics model based on DWI images to detect whether colorectal cancer patients have metastatic lesions within the scope of pelvic bone structure. Encouragingly, the model is robust and accurate in the prediction of test set, which can be used to undertake early warning of colorectal cancer bone metastasis and auxiliary diagnosis before treatment.

With the in-depth development of cross field artificial intelligence machine learning, it is now possible to predict disease risks through machines, and even diagnose some diseases (41). In recent years, due to the extensive application of deep learning and the inclusion of rich and diverse medical images, it has become an important part of artificial intelligence machine learning diagnosis, so deep learning has also had a huge impact in medical diagnosis. Previous studies have shown that the random forest algorithm can effectively process the mixed data, missing values or outliers, and higher dimensional data in medical data, and then comprehensively classify the data through multiple decision trees, and perform correlation testing, prediction, and interpretation (42). These processing processes are not easy to appear over fitting, making the prediction accuracy more accurate. In this study, we built and validated the prediction model of colorectal cancer bone metastasis through a large sample size, among which the prediction model established through the random forest (iterative) algorithm was the best (AUC: 0.926,95% CI: 0.873-0.979). In addition, the prediction efficiency of other machine learning models (ANNM, DTM, SVMM) is also better than GLRM. The possible reason is that Logistic regression has advantages in data processing of online relationships, and machine learning is often more applicable in the face of nonlinear problems. Therefore, how to improve the extrapolation of the model needs further research.

In addition, this study inevitably has the following limitations. First of all, this study only judged whether there was bone metastasis in the pelvic cavity at the patient level, and did not discuss the bone structure of a single pelvic cavity or from the focus level. In the future, we should also detect the metastatic lesions at the bone structure level and the lesion level, so as to detect and locate the bone metastasis of colorectal cancer in the pelvic region; Second, this study did not compare the classification performance of the radiomics model with the diagnostic efficacy of radiologists. In the follow-up research, we will compare the effectiveness of artificial intelligence and human experience; Third, this study only used a single DWI sequence to classify whether there is bone metastasis. Although this sequence is essential in the process of bone metastasis detection, it still has some limitations in the detection of osteogenic changes. Therefore, we consider adding other sequences (such as ADC map, T1WI, etc.) to the model in subsequent studies to improve the prediction performance of the model for all types of metastatic lesions.

## Conclusion

To sum up, this study based on depth learning segmentation DWI image pelvic bone structure of the radiomics model can better identify the pelvic range of colorectal cancer bone metastases; Among them, the predictive factors provided by RFM combined with GLCM can obtain the best predictive efficacy of bone metastasis, so it can undertake part of the work of mpMRI assisted diagnosis of colorectal cancer, so as to better guide clinical diagnosis and treatment.

## Data availability statement

The original contributions presented in the study are included in the article/**Supplementary Material**. Further inquiries can be directed to the corresponding author.

## Ethics statement

This retrospective study was approved by the Ethics Committee of Gezhouba Central Hospital of Sinopharm, and the research scheme was implemented according to the artificial intelligence(AI) model training specifications of the unit(No. 2020-006). In the informed consent statement of the patient involved in this article. As for oral informed consent, it generally means that if the patient’s education level is low or he cannot sign in person(due to either low education level or an inability to sign), the legal guardian designated by the patient will sign on his behalf, while the patient himself has orally declared his informed consent. When we signed this Informed Consent Form, for patients with oral informed consent, it was generally signed by the patient himself and the patient’s guardian at the same time, so there was no objection.

## Author contributions

JJ conceived and designed the study and wrote the manuscript. HZ, SS, ZT, HR, JF and XJ collected the data, performed the data analysis, and interpreted the outcome. All authors contributed to the article and approved the submitted version.
