# Supplementary material for: Machine learning based gray-level co-occurrence matrix early warning system enables accurate detection of colorectal cancer pelvic bone metastases on MRI
Source: Front Oncol. 2023 Mar 22;13:1121594. doi: 10.3389/fonc.2023.1121594 (PMC10073745; doi:10.3389/fonc.2023.1121594)
Supplement: Supplementary file 1 [file Image_1.pdf]

A

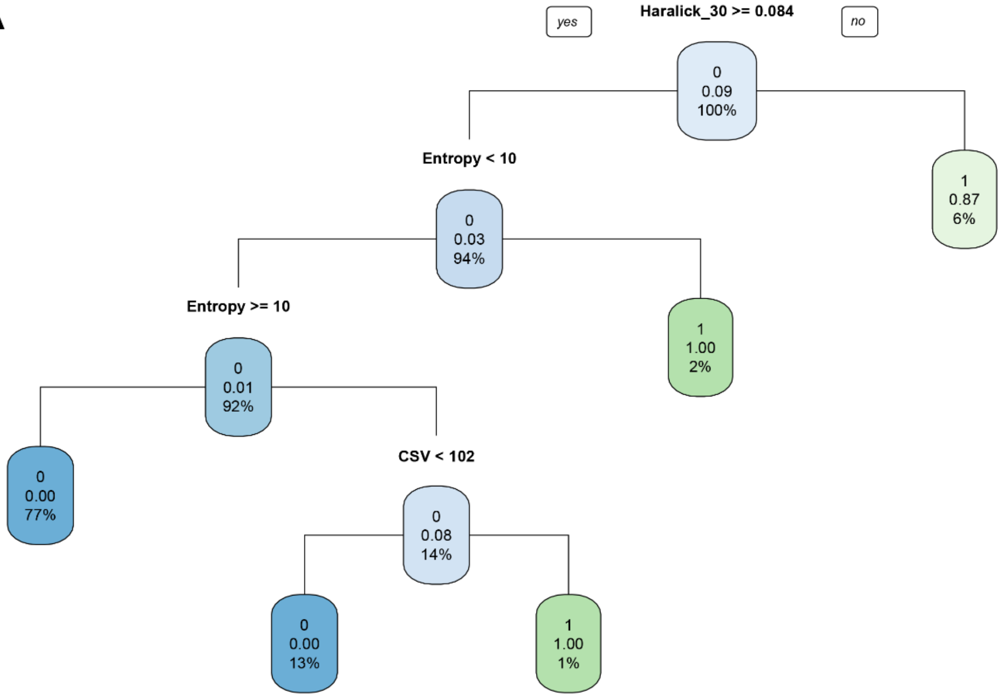

B

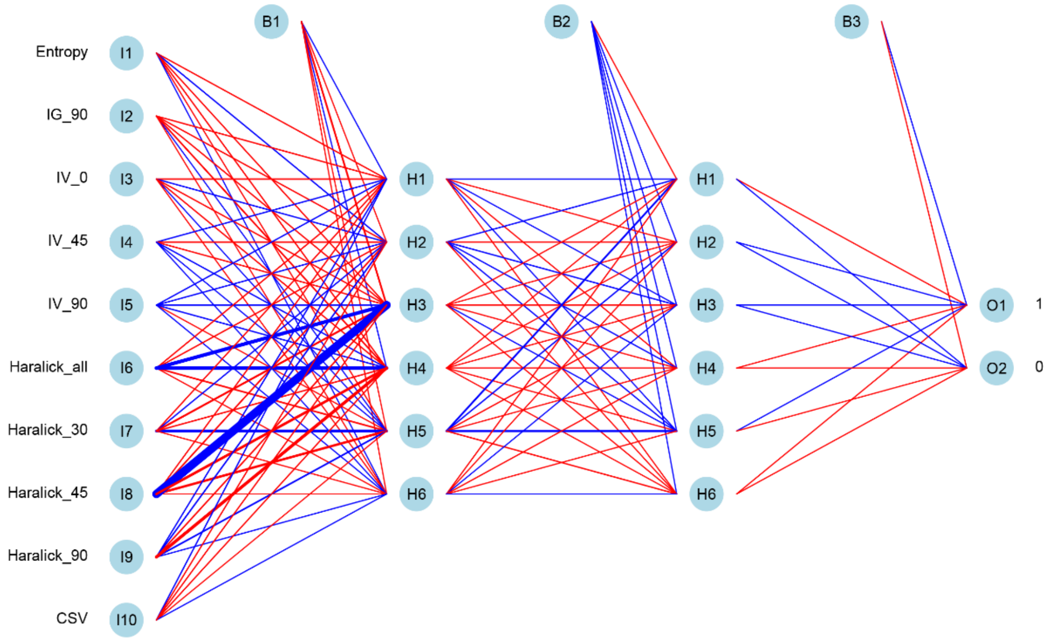

Supplementary Figure1. Visualization of pelvic bone metastasis prediction model based on different machine learning algorithms. A. DTM; B.ANNM.
