# Supplementary material for: Machine learning based gray-level co-occurrence matrix early warning system enables accurate detection of colorectal cancer pelvic bone metastases on MRI
Source: Front Oncol. 2023 Mar 22;13:1121594. doi: 10.3389/fonc.2023.1121594 (PMC10073745; doi:10.3389/fonc.2023.1121594)
Supplement: Supplementary file 3 [file Image_3.pdf]

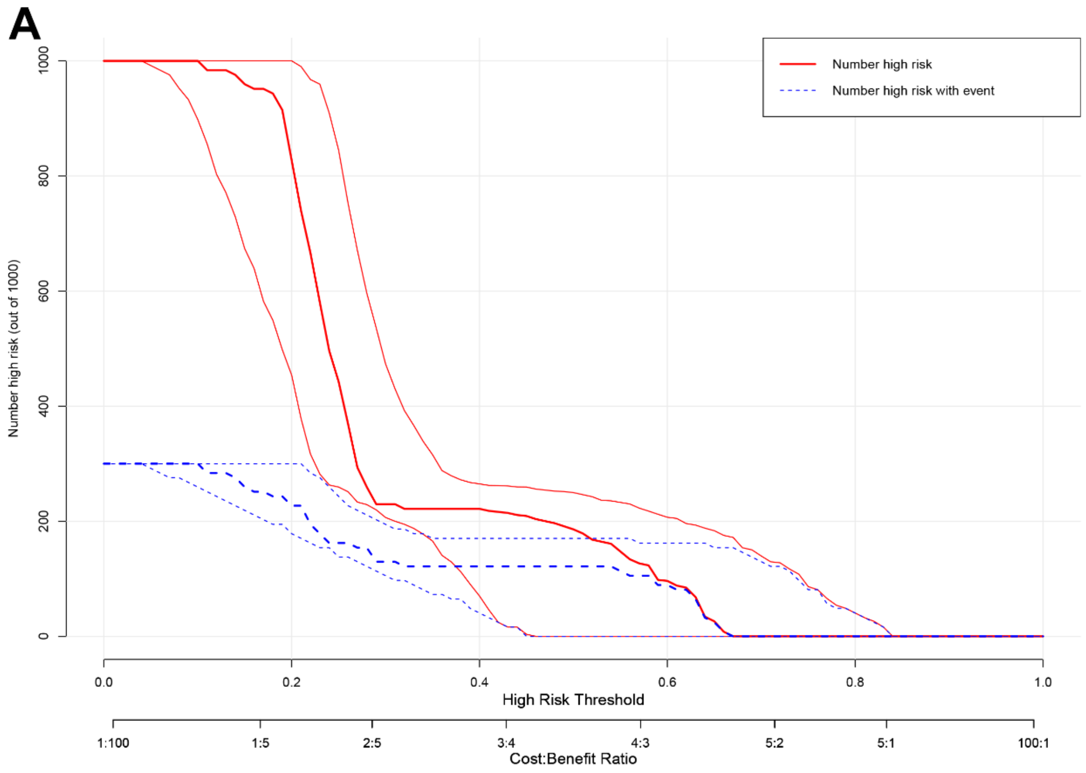

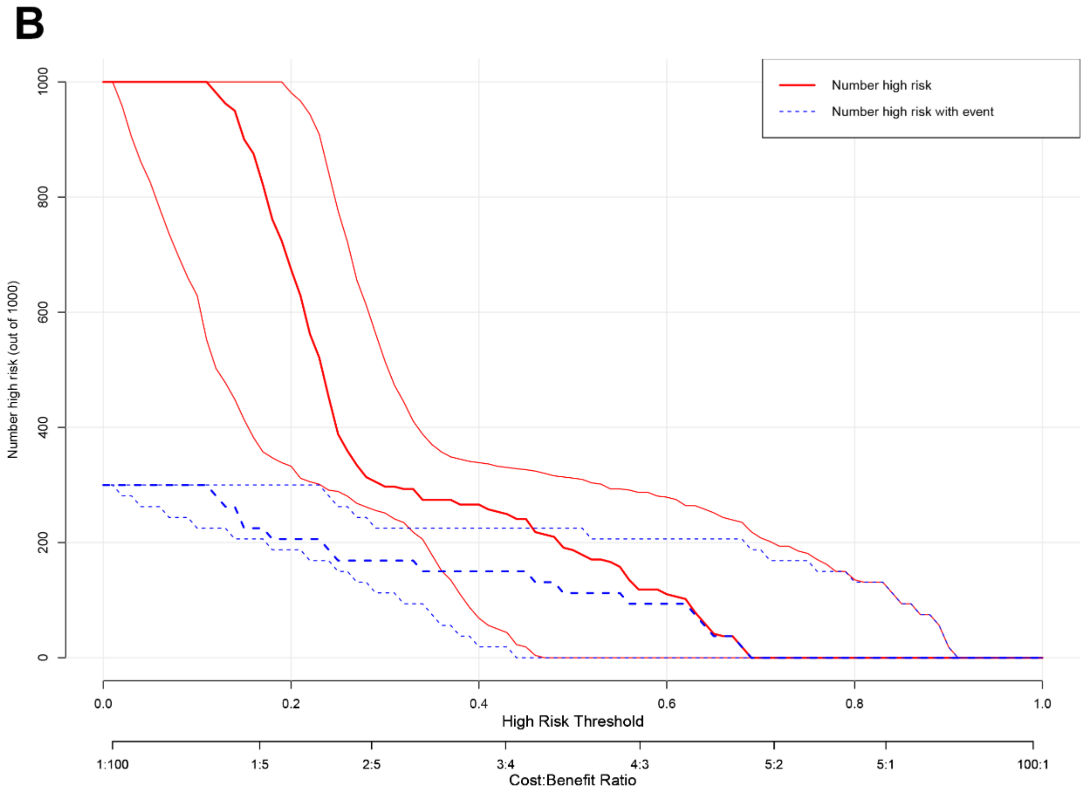

Supplementary Figure3. Identification ability of patients with bone metastasis based on CIC evaluation of RFM.A. Training Set;B. Internal validation set.
