# Supplementary material for: Machine learning based gray-level co-occurrence matrix early warning system enables accurate detection of colorectal cancer pelvic bone metastases on MRI
Source: Front Oncol. 2023 Mar 22;13:1121594. doi: 10.3389/fonc.2023.1121594 (PMC10073745; doi:10.3389/fonc.2023.1121594)
Supplement: Supplementary file 4 [file Table_1.docx]

Supplementary Table1. Comparative analysis of baseline data of pelvic bone metastasis and non bone metastasis in patients with colorectal cancer

| Variables | Training cohort | | | P-value | Validation cohort | | | P-value |
| --- | --- | --- | --- | --- | --- | --- | --- | --- |
|  | Overall(N=429) | Yes(N=37) | No(N=392) |  | Overall(N=185) | Yes(N=16) | No(N=169) |  |
| Age (median [IQR]) | 58.00 [50.00, 68.00] | 55.00 [47.00, 65.00] | 58.00 [50.00, 68.00] | 0.279 | 59.00 [49.00, 69.00] | 57.50 [54.00, 63.00] | 59.00 [49.00, 70.00] | 0.538 |
| sex (%) |  |  |  |  |  |  |  |  |
| Male | 261 (60.8) | 23 (62.2) | 238 (60.7) | 1 | 120 (64.9) | 13 (81.2) | 107 (63.3) | 0.245 |
| Female | 168 (39.2) | 14 (37.8) | 154 (39.3) |  | 65 (35.1) | 3 (18.8) | 62 (36.7) |  |
| Pathology (%) |  |  |  |  |  |  |  |  |
| Adenocarcinoma | 208 (48.5) | 12 (32.4) | 196 (50.0) | 0.134 | 85 (45.9) | 6 (37.5) | 79 (46.7) | 0.249 |
| Squamous cell carcinoma | 165 (38.5) | 17 (45.9) | 148 (37.8) |  | 66 (35.7) | 6 (37.5) | 60 (35.5) |  |
| Adenosquamous carcinoma | 40 (9.3) | 5 (13.5) | 35 (8.9) |  | 21 (11.4) | 1 (6.2) | 20 (11.8) |  |
| Small cell carcinoma | 16 (3.7) | 3 (8.1) | 13 (3.3) |  | 13 (7.0) | 3 (18.8) | 10 (5.9) |  |
| Tumor_stage (%) |  |  |  |  |  |  |  |  |
| I-II | 372 (86.7) | 5 (13.5) | 367 (93.6) | <0.001 | 161 (87.0) | 2 (12.5) | 159 (94.1) | <0.001 |
| III | 26 (6.1) | 13 (35.1) | 13 (3.3) |  | 13 (7.0) | 9 (56.2) | 4 (2.4) |  |
| IV | 31 (7.2) | 19 (51.4) | 12 (3.1) |  | 11 (5.9) | 5 (31.2) | 6 (3.6) |  |
| Differentiation (%) |  |  |  |  |  |  |  |  |
| High | 228 (53.1) | 5 (13.5) | 223 (56.9) | <0.001 | 98 (53.0) | 0 (0.0) | 98 (58.0) | <0.001 |
| Moderate | 134 (31.2) | 5 (13.5) | 129 (32.9) |  | 55 (29.7) | 3 (18.8) | 52 (30.8) |  |
| Low | 67 (15.6) | 27 (73.0) | 40 (10.2) |  | 32 (17.3) | 13 (81.2) | 19 (11.2) |  |
| OA (%) |  |  |  |  |  |  |  |  |
| Osteolytic | 278 (64.8) | 19 (51.4) | 259 (66.1) | 0.191 | 115 (62.2) | 7 (43.8) | 108 (63.9) | 0.224 |
| Osteogenic | 129 (30.1) | 15 (40.5) | 114 (29.1) |  | 59 (31.9) | 7 (43.8) | 52 (30.8) |  |
| Miscibility | 22 (5.1) | 3 (8.1) | 19 (4.8) |  | 11 (5.9) | 2 (12.5) | 9 (5.3) |  |
| CEA (%),ng/mL |  |  |  |  |  |  |  |  |
| ＜100 | 113 (26.3) | 7 (18.9) | 106 (27.0) | 0.381 | 43 (23.2) | 2 (12.5) | 41 (24.3) | 0.45 |
| ≥100 | 316 (73.7) | 30 (81.1) | 286 (73.0) |  | 142 (76.8) | 14 (87.5) | 128 (75.7) |  |
| Tumor_location (%) |  |  |  |  |  |  |  |  |
| Colonic segment | 145 (33.8) | 14 (37.8) | 131 (33.4) | 0.718 | 52 (28.1) | 3 (18.8) | 49 (29.0) | 0.562 |
| Rectal segment | 284 (66.2) | 23 (62.2) | 261 (66.6) |  | 133 (71.9) | 13 (81.2) | 120 (71.0) |  |
| ECOG (%) |  |  |  |  |  |  |  |  |
| 0-2 | 187 (43.6) | 30 (81.1) | 157 (40.1) | <0.001 | 91 (49.2) | 13 (81.2) | 78 (46.2) | 0.015 |
| ＞2 | 242 (56.4) | 7 (18.9) | 235 (59.9) |  | 94 (50.8) | 3 (18.8) | 91 (53.8) |  |
| EV (median [IQR]) | 0.91 [0.66, 1.21] | 0.83 [0.59, 1.12] | 0.92 [0.67, 1.21] | 0.252 | 1.00 [0.80, 1.23] | 0.93 [0.78, 1.05] | 1.01 [0.80, 1.24] | 0.176 |
| Entropy (median [IQR]) | 8.61 [8.36, 8.87] | 10.48 [10.11, 10.92] | 8.54 [8.35, 8.81] | <0.001 | 8.67 [8.44, 8.88] | 10.50 [10.27, 10.90] | 8.64 [8.42, 8.83] | <0.001 |
| IG_all (median [IQR]) | 3.06 [2.63, 3.60] | 3.23 [2.61, 3.78] | 3.04 [2.63, 3.60] | 0.443 | 3.04 [2.58, 3.52] | 2.95 [2.60, 3.41] | 3.05 [2.58, 3.52] | 0.82 |
| IG_0 (median [IQR]) | 2.21 [1.82, 2.61] | 3.82 [3.10, 4.04] | 2.12 [1.78, 2.52] | <0.001 | 2.20 [1.85, 2.52] | 3.46 [2.68, 3.89] | 2.14 [1.81, 2.45] | <0.001 |
| IG_45 (median [IQR]) | 2.95 [2.53, 3.39] | 3.07 [2.57, 3.53] | 2.95 [2.53, 3.38] | 0.408 | 3.02 [2.58, 3.39] | 3.32 [2.76, 3.46] | 3.00 [2.55, 3.34] | 0.235 |
| IG_90 (median [IQR]) | 2.28 [1.80, 2.75] | 3.43 [2.92, 3.86] | 2.22 [1.76, 2.62] | <0.001 | 2.33 [1.88, 2.78] | 3.36 [3.18, 3.86] | 2.27 [1.79, 2.64] | <0.001 |
| IV_all (median [IQR]) | 187.00 [161.00, 216.00] | 181.00 [159.00, 203.00] | 188.00 [161.75, 217.00] | 0.396 | 186.00 [157.00, 217.00] | 209.50 [172.75, 224.00] | 186.00 [157.00, 216.00] | 0.194 |
| IV_all_SD (median [IQR]) | 5274.00 [3523.00, 6927.00] | 5062.00 [3886.00, 6935.00] | 5348.00 [3483.75, 6922.50] | 0.754 | 5218.00 [3954.00, 7092.00] | 6289.50 [5051.25, 6984.25] | 5031.00 [3951.00, 7092.00] | 0.258 |
| IV_0 (median [IQR]) | 160.60 [128.80, 194.00] | 105.80 [80.30, 124.20] | 164.85 [137.65, 198.43] | <0.001 | 153.10 [124.90, 192.60] | 86.90 [60.08, 127.80] | 159.80 [130.70, 194.90] | <0.001 |
| IV_45 (median [IQR]) | 159.90 [127.20, 190.20] | 143.90 [122.70, 175.40] | 161.85 [127.50, 191.62] | 0.147 | 159.30 [120.60, 196.00] | 161.75 [110.55, 203.78] | 159.30 [121.10, 195.90] | 0.934 |
| IV_90 (median [IQR]) | 131.00 [109.00, 154.00] | 109.00 [96.00, 124.00] | 134.00 [111.00, 156.25] | <0.001 | 132.00 [105.00, 158.00] | 97.00 [90.25, 115.00] | 139.00 [111.00, 161.00] | <0.001 |
| Haralick_all (median [IQR]) | 0.10 [0.09, 0.10] | 0.13 [0.12, 0.14] | 0.09 [0.09, 0.10] | <0.001 | 0.10 [0.09, 0.10] | 0.12 [0.11, 0.13] | 0.10 [0.09, 0.10] | <0.001 |
| Haralick_30 (median [IQR]) | 0.10 [0.09, 0.11] | 0.14 [0.13, 0.15] | 0.10 [0.09, 0.11] | <0.001 | 0.10 [0.09, 0.11] | 0.14 [0.13, 0.16] | 0.10 [0.09, 0.11] | <0.001 |
| Haralick_45 (median [IQR]) | 0.07 [0.07, 0.08] | 0.12 [0.10, 0.13] | 0.07 [0.07, 0.08] | <0.001 | 0.07 [0.07, 0.08] | 0.11 [0.10, 0.12] | 0.07 [0.07, 0.08] | <0.001 |
| Haralick_90 (median [IQR]) | 0.11 [0.10, 0.13] | 0.14 [0.12, 0.16] | 0.11 [0.09, 0.13] | <0.001 | 0.11 [0.10, 0.12] | 0.14 [0.12, 0.16] | 0.11 [0.10, 0.12] | <0.001 |
| CSV (median [IQR]) | 90.00 [84.00, 96.00] | 124.00 [113.00, 134.00] | 89.00 [83.75, 95.00] | <0.001 | 89.00 [85.00, 95.00] | 137.00 [126.75, 142.75] | 88.00 [84.00, 93.00] | <0.001 |
| CP (median [IQR]) | 79.00 [72.00, 85.00] | 78.00 [73.00, 82.00] | 79.00 [72.00, 85.00] | 0.244 | 78.00 [72.00, 85.00] | 74.50 [72.00, 78.00] | 79.00 [72.00, 85.00] | 0.151 |

Abbreviations: IQR. inter-quartile range; OA,osseous alteration; CEA, carcinoembryonic antigen; ECOG, Eastern Cooperative Oncology Group; EV. Energy value;IG_all. Inverse gap full angle;IG_0. Inverse gap 0°;IG_45. Inverse gap 45°;IG_90. Inverse gap 90°;IV_all. Inertia value full angle;IV_all_SD. Inertia value full angle SD;IV_0. Inertia value 0°;IV_45. Inertia value 45°;IV_90. Inertia value 90°;Haralick_all. Haralick full angle;Haralick_0. Haralick 0°; Haralick_30. Haralick 30°; Haralick_45. Haralick 45°;Haralick_90. Haralick 90°;CSV. Cluster shadow value;CP. Cluster prominence.
