# Supplementary material for: Machine learning based gray-level co-occurrence matrix early warning system enables accurate detection of colorectal cancer pelvic bone metastases on MRI
Source: Front Oncol. 2023 Mar 22;13:1121594. doi: 10.3389/fonc.2023.1121594 (PMC10073745; doi:10.3389/fonc.2023.1121594)
Supplement: Supplementary file 5 [file Table_2.docx]

Supplementary Table2. Weight value of candidate variables for predicting pelvic bone metastasis based on RFM algorithm.

| Variables | Mean decrease accuracy | Mean decrease Gini |
| --- | --- | --- |
| age | -7.56E-35 | 4.91E-15 |
| sex | -1.02E-35 | 5.85E-16 |
| pathology | 1.000500375 | 0.00195287 |
| tumor_stage | 2.410280649 | 0.199579705 |
| differentiation | 1.430566372 | 0.035387161 |
| OA | 1.19E-35 | 1.66E-15 |
| CEA | -1.11E-36 | 2.09E-16 |
| tumor_location | -1.74E-35 | 5.66E-16 |
| ECOG | 1.000500375 | 0.002803977 |
| EV | -1.000500375 | 0.010938259 |
| Entropy | 19.26722742 | 11.13731126 |
| IG_all | -1.90E-21 | 0.002102579 |
| IG_0 | 9.533438974 | 2.021174162 |
| IG_45 | 3.08E-36 | 6.49E-15 |
| IG_90 | 8.588676232 | 1.899900606 |
| IV_all | 1.41546058 | 0.009267057 |
| IV_all_SD | -1.000500375 | 0.002481618 |
| IV_0 | 5.670518752 | 0.573914923 |
| IV_45 | 1.000500375 | 0.003115884 |
| IV_90 | 1.58259541 | 0.019431568 |
| Haralick_all | 11.79833896 | 4.612747302 |
| Haralick_30 | 11.59138936 | 4.506812978 |
| Haralick_45 | 20.09261753 | 12.30344847 |
| Haralick_90 | 3.863127183 | 0.193753591 |
| CSV | 18.69895807 | 10.92159455 |
| CP | -2.04E-21 | 0.000542063 |
