# Supplementary material for: Machine learning based gray-level co-occurrence matrix early warning system enables accurate detection of colorectal cancer pelvic bone metastases on MRI
Source: Front Oncol. 2023 Mar 22;13:1121594. doi: 10.3389/fonc.2023.1121594 (PMC10073745; doi:10.3389/fonc.2023.1121594)
Supplement: Supplementary file 6 [file Table_3.docx]

Supplementary Table3. Threshold probability of DCA for five types of pelvic bone metastasis prediction models

| **Model** | **thresholds** | **NB** |
| --- | --- | --- |
| RFM | 0.81 | 0.95 |
| SVMM | 0.80 | 0.83 |
| DTM | 0.81 | 0.86 |
| ANNM | 0.82 | 0.91 |
| GLRM | 0.54 | 0.72 |

Abbreviations: NB. Net benefit.
